# Died

**Published:** 1882-10

**Authors:** 


### Died.

In New York City, Aug. 31st, Belle, youngest daughter of Dr. Corydon Palmer, of Warren, Ohio.

It was with feelings of sadness and pain that we read the above notice in a morning paper a short time ago.

We had met Miss Palmer but a few times, yet we knew well her worth, and her gentle, loving disposition had endeared her to her circle of friends in an unusual degree. By nature, retiring and sensitive, those who knew her best, loved her most.

Dr. Palmer and his bereaved family have the sympathy of all their acquaintances and friends for this gentle daughter and sister, who has gone before.

